# M. Dupuytren on Varicose Aneurism, with Criticisms

**Published:** 1830-10-01

**Authors:** 


### XXXIV.

### M. Dupuytren on Varicose Aneurism.*

There are two duties imposed on the

* Repertoire d'Anatomie. Tome 8.

medical journalist. The first is to announce to the public what is new, and the second, equally, nay more important, to remind them from time to time of what is true, although it may want the smooth varnish of novelty. If the son of David was right when he said that nothing new could be found beneath the sun, it is obvious that the man who lives only on such provender must starve; and we think we may venture to affirm, that the accuracy of the Hebrew Monarch is more quickly apparent to none than the editors of medical journals. They speedily discover the nothingness of discoveries, and find that the novel ideas of our teeming and prolific periodical contributors are not unlike the produce of Suffolk dairies—treble skimm'd sky blue.

We were led to these disheartening reflections by the perusal of a memoir on varicose aneurism, from the pen of M. Dupuytren. It is ushered in with a pompous flourish by our able confrères of the Répertoire, but we really deem it more indicative of judgment and good sense than of any extraordinary ingenuity or talent. We think we shall be doing no disservice to the English reader by putting him in possession of the opinions and experience of the eminent surgeon of the Hôtel Dieu. His observations are communicated in the form of a letter to M. Husson, M.D. on the case of a young gentleman, who had been wounded eighteen months previously by a fowling-piece, charged with small shot. These had passed through the shoulder from before backwards, about the height of the cervix humeri, and a varicose aneurism in the right axilla was the consequence. It is amusing to find, that M. Dupuytren, who has lately been accused of robbing John Hunter of the merit of originating the operation for aneurism now in use, attributes to him on this occasion an act to which he can lay no claim. It was not he, but Dr. William Hunter, who first gave a description of the aneurismal varyx, in the Medical Observations and Inquiries. Before we revert to the subject matter of the memoir, we must take the opportunity of making one remark. When the puncture of the artery and vein communicate directly and immediately with each other, no pouch or sac intervening between them, the affection is called "aneurismal varyx." This was what was originally described by Dr. Hunter, but he afterwards pointed out a variety of this affection, in which a sac of aneurismal character, and formed from the cellular membrane, is placed between the wounded vessels. Later writers have designated this a varicose aneurism.

Now this was the case in M. Dupuytren's patient. There was present a small, round pulsating tumour between the artery and vein, accompanied with dilatation of the axillary veins, and a thrilling noise produced by the impulse of arterial blood into them. From the situation of the wound it would appear that the vessels had been wounded high up, but on accurate examination M. Dupuytren ascertained, that this was not the case. The shot had entered them near their termination in the brachial division, a circumstance probably occasioned by the position of the limb at the time of the accident. M. Dupuytren considers seriatim, the modes of treatment admissible in such a case. A question immediately presents itself to the surgeon:—is the disease productive of much inconvenience, or is it likely to prove dangerous? In the present case, the inconvenience is slight, being confined to a little weakness and swelling of the limb. With regard to future consequences, M. Dupuytren believes that if the patient observes moderation in diet, and in other points, little need be apprehended. If mischief should occur, it would then be time to think of remedial measures.

Of these the first on the list is compression. In general this is difficult and painful; but in M. Dupuytren's case impracticable, from the situation of the artery enveloped in the axillary plexus of nerves. Independently of this, compression would be improper in a case of old standing, as the opening between the arteryand vein becomes rounded, and almost incapable of uniting at its edges.

The same objection lies against the ligature of the artery above the disease. M. Dupuytren declares that it *frequently* succeeds in recent varicose aneurism, but almost always fails when performed at a late period. In the former, the edges of the opening are not indisposed to inflammation and union, and the temporary stoppage of the circulation by means of the ligature on the artery above, allows of the completion of those necessary processes. In the latter, the edges being indisposed to unite, the blood gets round into the artery below the ligature, by means of the anastomotic channels, and from thence it passes through the opening into the vein and intermediate sac. The pulsation of the latter, and the dilatation, &c. of the vein subside for a short time after the operation, but generally reappear in three or four days. The facility for the reflux of the blood in the varicose aneurism is much greater than that afforded by ordinary aneurism for reasons sufficiently obvious. In order to place a varicose aneurism in the same condition by an operation, as the common aneurism is after tying the artery above, it is necessary not only to do this, but likewise to tie the vein above and below the opening in its side. This, however, has never been attempted.

M. Dupuytren believes that the coexistence of a sac with the arterio-venous communication renders the chances of success from the Hunterian operation greater, in as much as the disease approaches nearer in character to ordinary aneurism. He thinks notwithstanding that it would be safer to apply a ligature above and below the aneurismal sac. Supposing it is determined to operate in this manner, two plans will present themselves to the consideration of the surgeon. Either the two ends of the artery may be tied at the same time, by opening into the aneurismal sac, or the latter may be left untouched, and the vessel tied alone with two separate ligatures. The former is attended with many inconveniences, with difficulty, delay, and even danger. The blood flows into the sac through the venous opening, fills up the wound, and embarrasses the operator. The lower part of the artery should be first secured, in order that the pulsations may still direct the operator to the upper portion. In the other operation the inferior end of the vessel is at once cut down upon and tied without any intermeddling with the sac, and then the superior is treated in the same manner. M. Dupuytren concludes by adverting to an operation to which we before alluded, viz. tying the upper portion of the artery, and applying a ligature to the *vein* above and below the wound in it. To this he appears to hint a preference, above the other modes of proceeding. With reference to the particular case on which the foregoing remarks were founded, it is to be understood that M. Dupuytren advises nothing in the way of operation to be attempted, till urgent symptoms shall arise to require one.

We have given a faithful abrégé of M. Dupuytren's sentiments, without interrupting the tenour of their course by comment of our own. We must say that we doubt the propriety of the operation of tying the vein. Superficial veins, like the saphena or those of the upper extremity, are extremely prone to fatal inflammation; a danger sufficiently attested by the general abandonment, in this country, of the different operations for varices. This we think is a valid argument against M. Dupuytren's proposal.

The opinions entertained by British surgeons, on the treatment of varicose aneurism or aneurismal varyx, may be shortly stated as follows. If the disease shows no disposition to increase, and it usually does not, no operative measures are required. Scarpa, Hunter, Bell, and others mention cases of the aneurismal varyx that remained stationary for a great number of years. Compression has been tried, and in several cases it is said to have succeeded. Under favourable circumstances, we should think that moderate compression would be highly proper in the early stage of the complaint. Two cases are recorded in the Medical Facts and Observations, in which the varyx being complicated with aneurism that threatened bursting, the sacs were opened, and a ligature placed on the artery above and below the aperture in it. If an operation were rendered indispensable by the increase of the sac, or other circumstances, we should prefer tying the artery above and below the sac, without making an incision into latter. Such we believe to be the present extent of our information on this subject.

---

